# Yttrium Trifluoride as a Marker of Infiltration Rate of Decalcified Root Cementum: An In Vitro Study

**DOI:** 10.3390/polym14040780

**Published:** 2022-02-17

**Authors:** Anna Nowak-Wachol, Anna Korytkowska-Wałach, Bartosz Chmiela, Kacper Wachol, Maciej Łopaciński, Magdalena Wyszyńska, Yousuf Al-Dulaimi, Małgorzata Skucha-Nowak

**Affiliations:** 1 Department of Dental Propedeutics, Division of Medical Sciences in Zabrze, Doctoral School, Medical University of Silesia in Katowice, 15 Poniatowskiego Street, 40-055 Katowice, Poland; 2 Department of Organic Chemistry, Bioorganic Chemistry and Biotechnology, Faculty of Chemistry, Silesian University of Technology, ul. Krzywoustego 4, 44-100 Gliwice, Poland; anna.korytkowska-walach@polsl.pl; 3 Department of Materials Technologies, Faculty of Materials Engineering, Silesian University of Technology, ul. Krasińskiego 8, 40-019 Katowice, Poland; bartosz.chmiela@polsl.pl; 4 Department of Oral Surgery, Division of Medical Sciences in Zabrze, Medical University of Silesia, 15 Poniatowskiego Street, 40-055 Katowice, Poland; kacper.wachol@sum.edu.pl; 5 Department of Periodontal and Oral Mucosa Diseases, Division of Medical Sciences in Zabrze, Medical University of Silesia, 15 Poniatowskiego Street, 40−055 Katowice, Poland; mlopacinski@sum.edu.pl; 6 Department of Dental Material Sciences, Division of Medical Sciences in Zabrze, Medical University of Silesia, 15 Poniatowskiego Street, 40−055 Katowice, Poland; magdalena.wyszynska@sum.edu.pl; 7 5th Year Dentistry Program, Student Scientific Society in Department of Prosthetic Dentistry and Dental Material Sciences, Division of Medical Sciences in Zabrze, Medical University of Silesia, 15 Poniatowskiego Street, 40−055 Katowice, Poland; yousifaldulaimi1@gmail.com; 8 Department of Dental Propedeutics, Division of Medical Sciences in Zabrze, Medical University of Silesia in Katowice, 15 Poniatowskiego Street, 40-055 Katowice, Poland; mskucha-nowak@sum.edu.pl

**Keywords:** root caries, resin infiltration, polymers, minimally invasive dentistry

## Abstract

Research related to the development of a dental infiltrant for minimally invasive treatment of initial caries of hard dental tissues is presented. The formulation of the developed infiltrant material includes typical methacrylate monomers used in dentistry, an author’s adhesion monomer containing metronidazole, a photoinitiating system and yttrium trifluoride (YF_3_). The main objective of the study was to evaluate penetration into decalcified root cementum using scanning electron microscope of an experimental preparation with the characteristics of a dental infiltrant compared to a commercial preparation with the addition of YF_3_ as a contrast agent. Microscopic observations showed that YF_3_ particles virtually did not penetrate deep into the root cementum—this was mainly due to the particle size of YF_3_. Observations of cementum and root dentin tissue infiltration: resin tissue infiltration was visible to a depth of about 80 to 120 μm without the use of a tracer, which, due to agglomeration and particle size, remained on the cementum surface or in the resin used for inlaying. There were no differences between the degree of penetration of an experimental preparation with the characteristics of a dental infiltrant, as compared to a commercial preparation.

## 1. Introduction

Root cementum (Latin: *cementum*), a mineralised tissue covering the entire root surface of a human tooth, is part of the marginal periodontium which connects the tooth to the alveolar bone through the connective tissue ligaments of the periodontium. The primary function of root cementum is to secure the tooth in the alveolus. The first detailed descriptions of the dental anatomy and microscopic observations of the root cementum structure were made by Fränkel and Raschkow (1835), and by Retzius (1836) [1]. In terms of chemical structure, it comprises inorganic and organic parts. The inorganic part, which consists of hydroxyapatite crystals, accounts for about 50% of the dry matter. The organic part mainly contains collagen and, to a lesser extent, glycoproteins and proteoglycans [2]. With regard to its structure, we can distinguish between the following: cell-free cementum, which directly covers dentin of the tooth root, and cellular cementum, which is found on the surface of cell-free cementum, primarily in the periapical region of the tooth root [3].

Root caries is the only form of caries found in indigenous populations in Papua New Guinea, and, in those communities, it is the main cause of tooth loss. A study of ancient skulls showed that caries within the root was more common than caries within the crown [4,5]. Caries as a disease process is nowadays an important problem of ageing societies in developed countries. It involves hard tissues of the tooth, including the root cementum (so-called cementum caries, caries of the tooth root—*caries cementumi, caries radicis dentis*). It occurs after root tissues are exposed due to recession or in the course of marginal periodontal disease and first affects cementum and then root dentin. Factors that modify its course include periodontal diseases leading to the exposure of the enamel–cementum junction, periodontal treatments, untreated caries of the tooth crown, removable prosthetic restorations, malocclusion, crowding of teeth, cavities of non-carious origin in the cervical region, xerostomia, Sjögren’s syndrome, diabetes, decreased salivary buffering capacity, decreased salivary immunoglobulin content, diet high in carbohydrates, and smoking. Deterioration of oral hygiene associated with reduced manual dexterity in the elderly also has a significant impact on its occurrence [6,7].

The carious process, which develops in the tooth root, is a soft and rapidly progressive lesion, limited to hard tissues; however, it may also undermine enamel at the border of the enamel–cementum junction. This disease, diagnosed according to International Caries Detection and Assessment system (ICDAS), classifies and detects root caries based on the following criteria: colour (light/dark brown/black); texture (smooth/rough); appearance (shiny/glossy, matte/nonglossy); perception on gentle probing (soft/leathery/hard); and cavitation (loss of anatomic contour) [8].

Minimally invasive dentistry aims at diagnosing and treating lesions at an early stage, while limiting surgical procedures within hard tissues and preserving the patient’s own tissues. One of the procedures of modern minimally invasive dentistry is infiltration of hard tooth. The infiltration procedure is a mode of treatment that bridges the gap between prevention and restoration of carious lesions of enamel and dentin, microscopically reaching up to 1/3 depth of dentin (D-1), reducing the visibility and masking white spots on the vestibular surfaces of teeth. It is a treatment method that fills, strengthens, and stabilises demineralised enamel without a loss of healthy tooth structures. The procedure involves infiltration of porous, decalcified enamel with a low-viscosity resin by means of capillary action, thus stopping the progression of lesions by closing the microporosities, which prevents diffusion of acids. The technique aims to create a diffusion barrier within the lesion rather than on the surface. Robinson et al. in their study showed that approximately 60 ± 10% of the pore volume was occupied by resin during the infiltration process [9]. According to Kielbassa et al., resin infiltrates carious lesions of subsurface enamel to a depth of over 100 µm [10].

Extensive research is still being carried out to reduce the risk of caries formation and to minimise its effects. Yttrium fluoride compounds are used in dentistry as antibacterial, anticarious preparations and to make the filling material visible on X-ray images (as a contrast). For the same purpose, elements such as barium, zinc, aluminium, strontium, zirconium, silicon, ytterbium, yttrium, and lanthanum are added to dental materials (e.g., composites) to make the radiopaque filler more absorbent of X-rays. Manufacturers of dental materials claim that the addition of yttrium fluoride provides impermeability to X-rays and released fluoride ions prevent development of secondary caries. Yttrium trifluoride composites include Pertac II, Filtek Silorane, Filtek P90, and Hytac compomer [11,12,13,14].

The infiltration procedure has so far been used to treat initial caries within enamel. The authors of this paper assumed that the infiltration method also seems feasible to prevent and treat initial caries of all hard tooth tissues, including root cementum. With this assumption in mind and with use of yttrium trifluoride, the authors synthesised an experimental preparation with the characteristics of a dental infiltrant [15]. An analogous experimental preparation containing yttrium trifluoride as one of the possible cariostatic and radioopaque agents has recently been filed for patent [16]. This preparation also has in its composition commercially available monomers commonly used in dental materials [triethylene glycol dimethacrylate (TEGDMA) and 2-hydroxyethyl methacrylate (HEMA)] and a monomer synthesized by attaching metronidazole (MTZ) to the adhesive monomer 5-(7-methyl-1,6-dioxo-2,5-dioxa-7-octenyl)trimellitic anhydride (PMMAn). The new PMMAn-MTZ monomer has adhesion ability to dentin and potential antimicrobial activity due to its content of metronidazole moiety. In the previous work, the synthesis of PMMAn was described. PMMAn was shown to exhibit good adhesion to dentin, which was confirmed by in vitro studies [17]. Recently, the properties of preparations in development containing PMMAn-MTZ have been described [15,18,19,20,21,22], while the synthesis of this monomer and characterization is presented for the first time.

Microscopic observation of the degree of infiltration of the preparation into the tooth tissue is difficult because it is colourless (transparent) and, after application, is the same colour as tooth tissue, which makes it difficult to determine the degree of its penetration into the decalcification area.

Null hypothesis: Following the results of our own research to date [15], for the purposes of this study the authors assumed that the addition of yttrium trifluoride as a contrast agent would allow for microscopic observations of the degree of penetration of the preparation into the decalcified tissue with the use of an X-ray micro-analyser.

The main objective of the study was to evaluate penetration into decalcified root cementum of an experimental preparation with the characteristics of a dental infiltrant when compared to a commercial preparation.

## 2. Materials and Methods

The *NMR spectra*: were recorded at 25 °C with the aid of 600 MHz Varian spectrometer in deuterated acetone. Tetramethylsilane (TMS) was used as an internal reference.

*Chemicals:* 5-(7-methyl-1,6-dioxo-2,5-dioxa-7-octenyl)trimellitate anhydride (PMMAn) was synthesized as previously reported [17]. Triethylene glycol dimethacrylate (TEGDMA, Fluka, Buchs, Switzerland), metronidazole (MTZ, Acros Organics, Antwerp, Belgium), camphorquinone (CQ, Aldrich, St. Louis, MO, USA), phenothiazyne (PTZ, Aldrich); all used as supplied. 2-hydroxyethyl methacrylate (HEMA, Sigma, St. Louis, MO, USA), *N,N*-dimethylaminoethyl methacrylate (DMAEMA, Merck, Rahway, NJ, USA), and acetone (POCh) were dried over anhydrous magnesium sulphate. Solvents and other auxiliary chemicals were of commercial grade.

*Synthesis of PMMAn-MTZ:* 0.500 g (1.4 mmol) PMMAn, 0.246 g (1.4 mmol) MTZ were dissolved in 28 mL of dried acetone and PTZ (inhibitor of radical polymerization, 100 ppm by weight in respect to reagents) was added. Then 14 μL DMAEMA (catalyst, 4% by weight in respect to the reagents) was added and the mixture was stirred magnetically at 40 °C for 5 days. The reaction was carried out in the dark in dried laboratory glassware (120 °C, 24 h). After completion of the reaction, acetone was evaporated to give the crude product, which was analysed by ^1^H NMR.

*Experimental preparation* was prepared in amber vial by mixing 0.05 g of PMMAn-MTZ, 3.75 g of TEGDMA and 1.25 g of HEMA. Then a photoinitiating system CQ/DMAEMA was added (0.5% and 1% respectively, of the total mass of the monomers). In order to thoroughly mix all components, the vial was placed on a magnetic stirrer and the mixing process was carried out for at least 15 min.

*Infiltration study:* The study material consisted of 10 human teeth—molars and premolars extracted due to periodontal reasons, with preserved anatomical crowns and roots without cavities and fillings in the cervical region. After extraction, the teeth were stored in a chloramine solution. The study group consisted of 6 demineralised teeth, while the control group (not subjected to the demineralisation process) consisted of 4 teeth. Before the onset of the study, the chloramine solution was poured off and the teeth were thoroughly rinsed in distilled water and left there for 24 h. After rinsing and drying, the teeth from the study group were subjected to a chemical decalcification process in a buffer solution which contained calcium chloride (3 mM/dm^3^), potassium dihydrogen phosphate(V) (3 mM/dm^3^), acetic acid (50 mM/dm^3^), methylenehydroxydiphosphonate [MHDP (6 M/dm^3^), and potassium hydroxide (in the amount sufficient to ensure that an appropriate pH level was obtained in the solution). Demineralisation was carried out at 37 °C for 4 weeks, while controlling and maintaining the pH below the level critical for the development of cementum and root dentin caries, that is 6.2 [15,18]. If necessary, the pH level was lowered by adding acetic acid.

After the decalcification process was completed, the teeth were rinsed 3 times in distilled water and left there for 24 h. Then the teeth were dried [19].

A commercially available preparation called Icon (DMG, Hamburg, Germany) and an experimental preparation with infiltrant characteristics were used in the study [20,21].

Yttrium trifluoride (nanopowder, 80–100 nm, Nanoshel LLC, Wilmington DE, USA) was added to both commercial and experimental preparations as an indicator of the depth of penetration ability into the demineralised cementum. Due to former experience of the authors’ own research, it was decided to add up to 2% YF_3_ and 4% YF_3_ [22]. The teeth from both test and control groups were divided into 2 groups (the test group consisted of 3 teeth and the control group of 2 teeth in each trial).

Group 1: 2% YF_3_

Group 2: 4% YF_3_

The teeth from both test and control groups were divided into 2 zones (A and B) by a red line drawn along the long axis of the tooth. In zone A, infiltration was carried out using the experimental preparation with YF_3_, in zone B—it was Icon with YF_3_. The preparations were applied according to the recommendations of the ICON manufacturer. During the polymerisation process, a LEDEX WL-070 lamp from Dentmeate was used.

**Figure 1:**
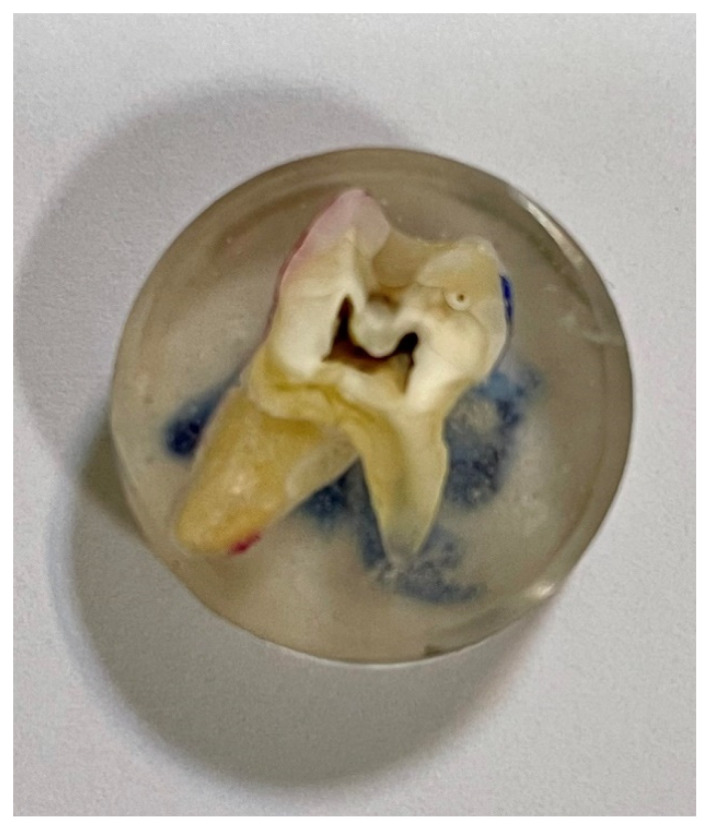
Specimen prepared for microscopic observation.

After soaking, the teeth were cold-inlaid in a two-component resin with low polymerisation shrinkage features (Figure 1). It was Metalogis Opti-Mix, based on methylmethacrylate (Metalogis s.c., Warsaw, Poland). Subsequently, the teeth were cut longitudinally with the use of a Buehler precision cutter (Buehler Holding A.G., Uzwil, Switzerland), after which they were cut to size. The cross-sectional surfaces were sanded with waterproof sandpaper of decreasing gradation P320, P500, P800, and P1000 Metalogis DEMPAX (Metalogis s.c, Warsaw, Poland); and then polished with diamond suspensions: Struers DP Suspension 9 μm, 3 μm, 1 μm, and 0.25 μm (Struers A/S, Ballerup, Denmark).

The study of tooth longitudinal section morphology and chemical composition was performed with the use of a Hitachi S-3400N scanning electron microscope (Hitachi Ltd., Tokyo, Japan) equipped with a Thermo Noran EDS energy dispersive X-ray spectrometer (Thermo Fisher Scientific Inc., Madison, WI, USA), under low vacuum conditions (50 Pa) using the backscattered electrons detector (BSE), at an accelerating voltage of 15 kV.

## 3. Results

**Figure 2:**
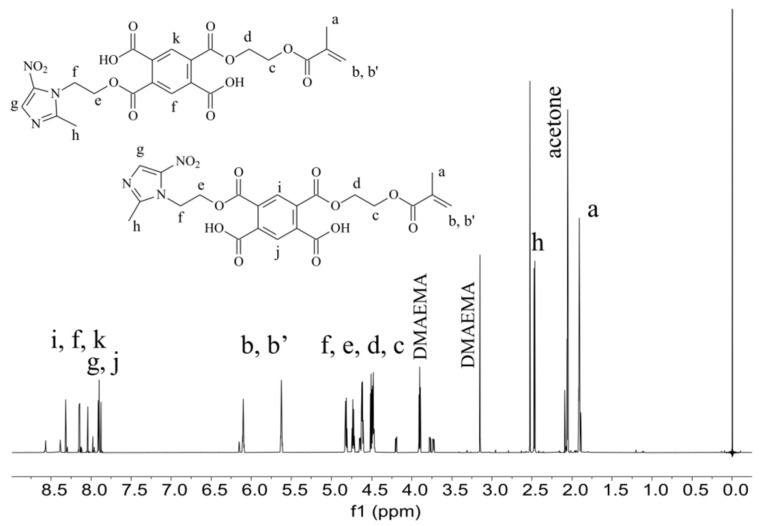
^1^H NMR spectrum of PMMAn-MTZ.

The experimental preparation contains TEGMMA and HEMA in the highest amount. These monomers are responsible for the formation of polymer network on the area of demineralized fragments of dental hard tissues in the process of polymerization. The formed polymeric material is designed to block the access of carious bacteria and their metabolic products destroying the structure of the hard dental tissues. Furthermore, the cured polymeric material provides a specific matrix for yttrium trifluoride embedded in it. The adhesive and antimicrobial agent (PMMAn-MTZ), added to the experimental preparation, is also incorporated during polymerization into the polymer network composed of TEGDMA and HEMA, described earlier. The PMMAn-MTZ monomer, the synthesis of which is described in this work, was characterized by 1H NMR (Figure 2) The signals seen on the spectrum indicate that the monomer obtained is a mixture of meta and para isomers (in a ratio of about 1:1). The characteristic signals of protons of the incorporated metronidazole are visible on the spectrum.

**Figure 3:**
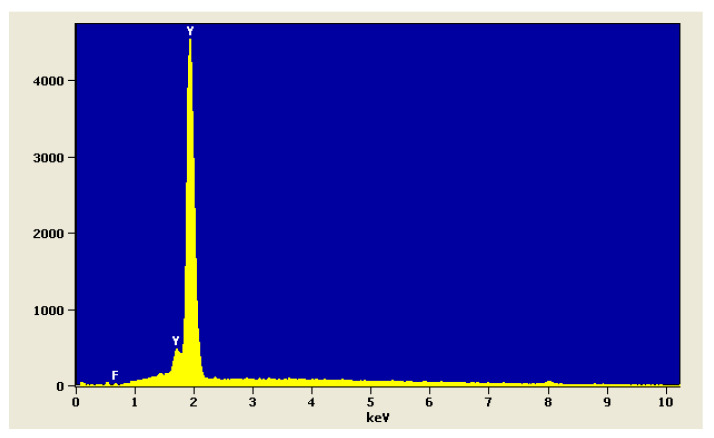
EDS spectrum of yttrium trifluoride powder.

The analysis of chemical composition of YF_3_ powder is shown in Figure 3.

The metronidazole attached to the compound should be released by the hydrolysis reaction of the ester bond between the metronidazole molecule and PMMAn, as a result of which the antimicrobial activity should persist for a long time.

**Figure 4:**
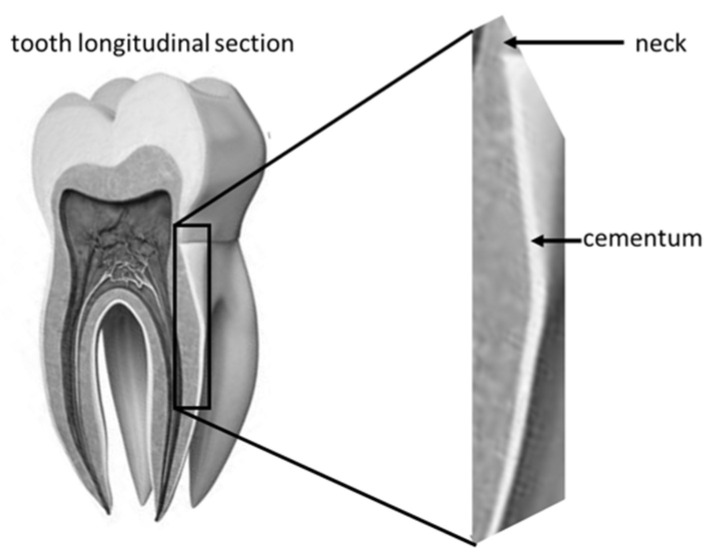
Tooth longitudinal section with highlighted area of SEM observations.

The examined area was root cementum, 4–6 mm below the tooth neck (Figure 4).

However, microscopic observations showed that yttrium fluoride particles virtually do not penetrate deep into the root cementum—this is mainly due to the particle size of YF_3_. The used tracer in the form of yttrium trifluoride agglomerates in most cases and remains in the form of conglomerates on the surface of the root cement. Single yttrium trifluoride particles can be seen to arise through the cementum tissues into the root dentin structure.

**Figure 5:**
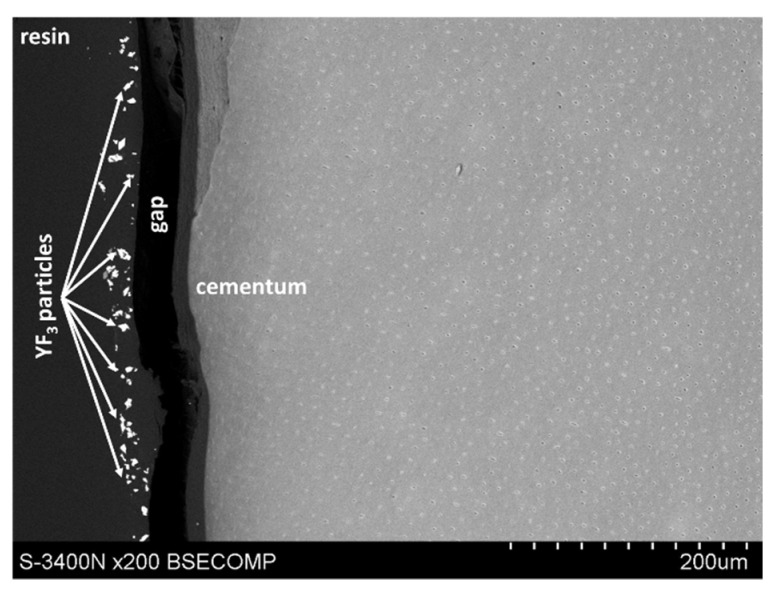
Icon + YF_3_ specimen sample: image of the longitudinal section of root cementum: YF_3_ particles in the mounting resin.

**Figure 6:**
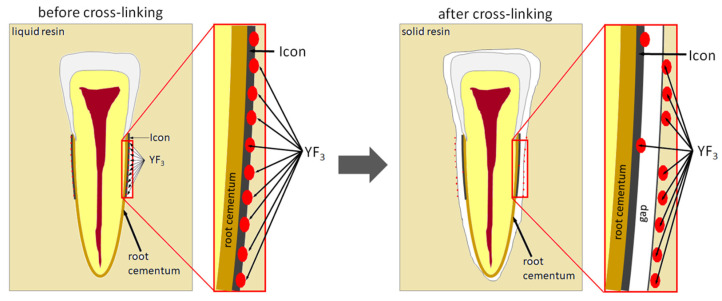
Scheme of displacement of yttrium particles after polymerisation of the embedding resin.

The study also showed that yttrium fluoride may have insufficient adhesion to the outer surface of tooth root cementum. Longitudinal sections of the teeth made after embedding them in resin showed large amounts of yttrium fluoride particles in the resin used to inlay specimens, sporadically on the surface of root cementum and deep into the cementum and dentin tissue of the root (Figure 5). This demonstrates high affinity of the yttrium fluoride preparation to the used resin (after completion of the crosslinking process, the resin shrinks slightly, forming a gap between the surface of root cementum and resin—Figure 6).

**Figure 7:**
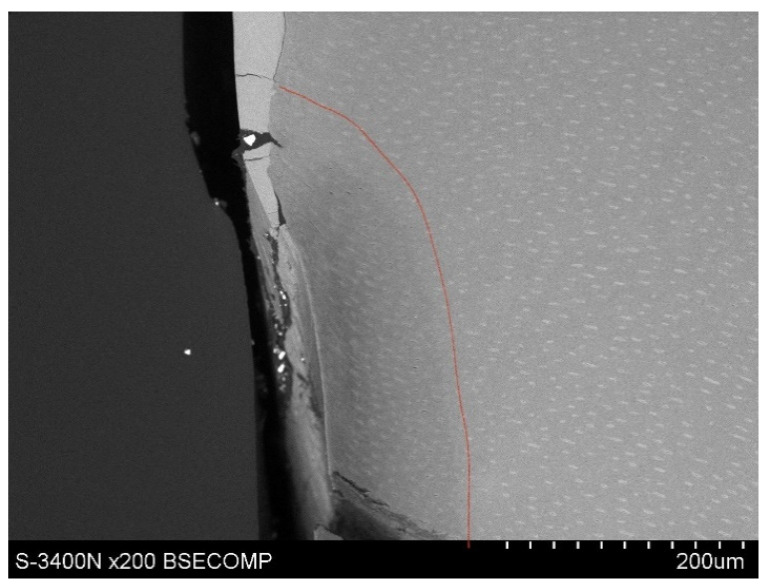
Polished section from the study group, Icon + 2% YF_3_, visible gradual brightening of the infiltrated root dentin, depth of infiltration at approx. 130 μm.

**Figure 8:**
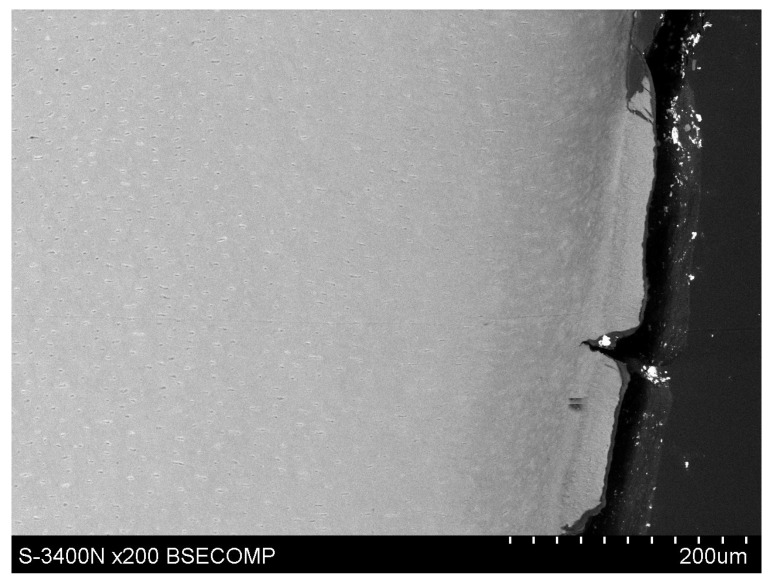
Polished section from the study group, experimental preparation + 4% YF_3_, visible infiltration of root dentin to 80–100 μm, visible large YF_3_ particle agglomerates in the inlaying resin.

**Figure 9:**
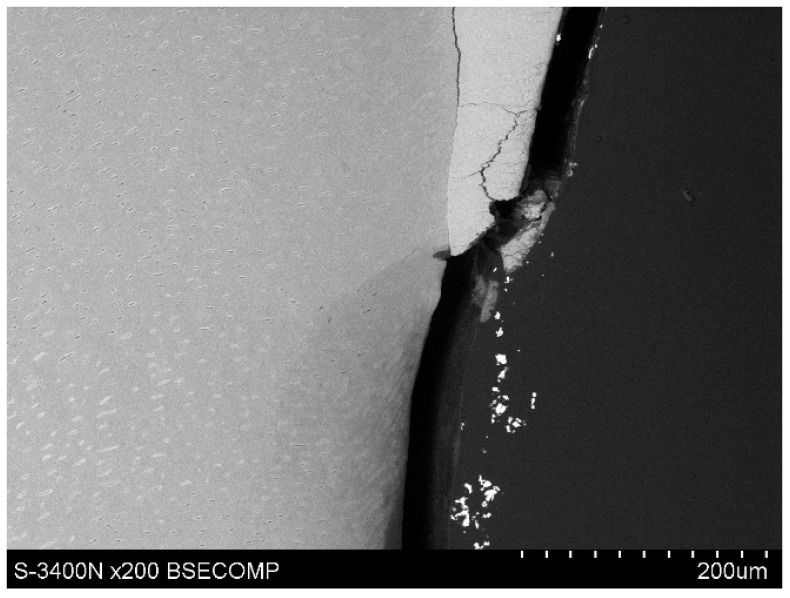
Polished section from the study group, Icon + 4% YF_3_, visible infiltration of the preparation to 100 μm of root dentin and large YF_3_ particle agglomerates in the inlaying resin.

**Figure 10:**
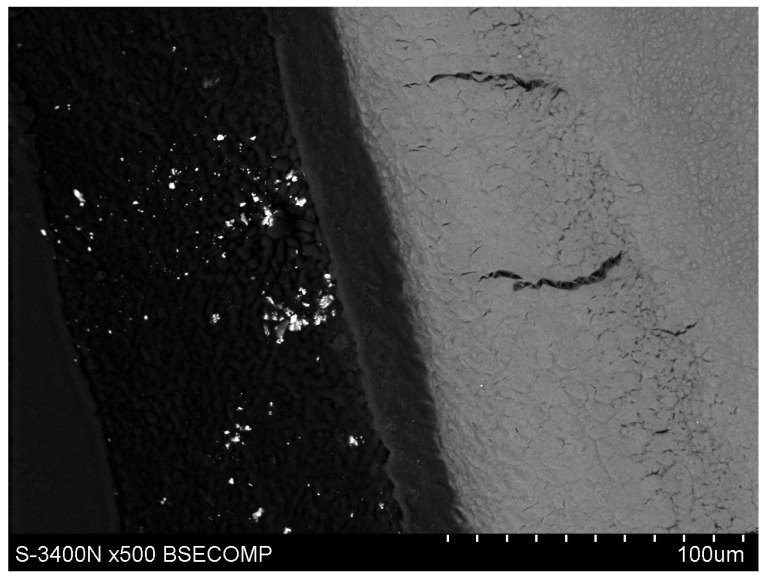
Polished section from the study group, experimental preparation + 2% YF_3_, visible single particles of yttrium trifluoride penetrating the tissue.

**Figure 11:**
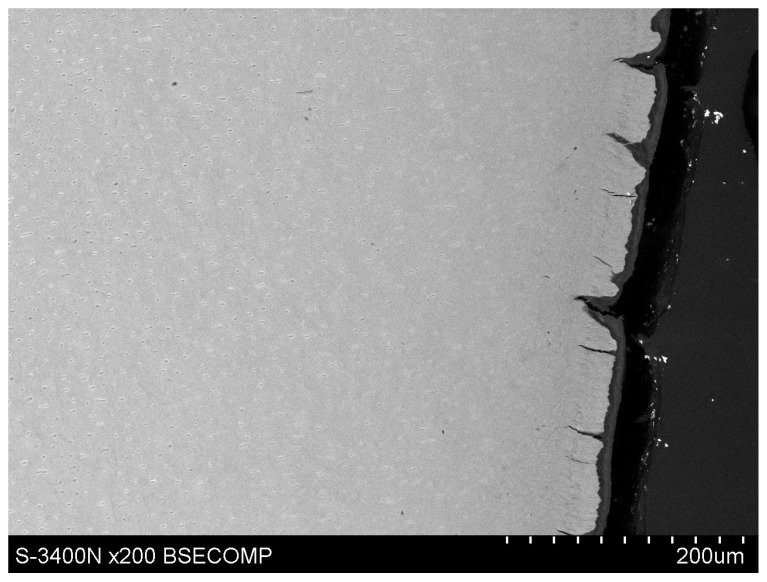
Polished section from the study group, experimental preparation + 4% YF_3_, visible single particles in cementum tissue and numerous YF_3_ agglomerates in the inlaying resin.

Observations of cementum and root dentin tissue infiltration—resin tissue infiltration is visible to a depth of about 80–120 μm without the use of a tracer, which due to agglomeration and particle size remained on the cementum surface or in the resin used for inlaying (Figure 7, Figure 8, Figure 9, Figure 10 and Figure 11).

## 4. Discussion

The incidence of caries on the root surface increases with age. A systematic review of dental caries incidence worldwide identified three peaks of activity—at the age of 6, 25, and 70 [23]. The peak incidence of root caries at the age of 70 is associated with exposed root surfaces due to recession and periodontal disease common in that age group. These surfaces are the site of bacterial biofilm deposition, as proper cleaning is difficult in the elderly due to reduced manual dexterity [24].

The treatment of root caries includes primary, secondary, and tertiary prevention. Primary prophylaxis includes procedures carried out before the onset of root caries, with the aim of preventing it. Secondary prevention of root caries focuses on changes early into the disease process, with the aim of inhibiting them. Tertiary prevention involves restorative treatment of root caries cavities to prevent complications. The traditional approach to the treatment of root cavities is based, among other things, on the Billings’ index and systematises treatment into conservative and invasive [25].

Primary and secondary prevention includes control of dietary carbohydrate intake, improved oral hygiene, antimicrobial agents, toothpastes that contain fluoride or arginine, fluoridated water, salt or milk; professionally applied topical fluoride (gels, varnishes, silver diamine fluoride solutions, etc.); amorphous calcium phosphate and casein phosphopeptide (ACP-CPP); and use of ozone [26].

Studies show that the results of fluoride treatment are dose-dependent. A laboratory study showed that fluoride at a concentration of 5000 ppm used in toothpaste was more effective at controlling the onset and progression of root caries than fluoride concentrations of 1300 or 1500 ppm [27]. Furthermore, toothpastes with higher fluoride concentrations (5000 and 2800 ppm) improved the acid resistance of bovine root dentin more effectively than at 1450 ppm [28]. The properties of dose-dependent antimicrobial effect of fluoride were also confirmed in a clinical study in which a 5000 ppm fluoride toothpaste was more effective in controlling the progression of root caries lesions among the elderly than ordinary toothpastes with 1000–1450 ppm content of fluoride [29,30].

The most common ways to prevent and treat initial root caries are remineralisation with preparations containing fluoride and use of antimicrobial preparations which include chlorhexidine or triclosan. Another way to prevent root caries is to seal the root surface with glutaraldehyde, which is a fixative and a component of some adhesive systems. It was shown that sealing the root surface with two types of dentin adhesive systems which contained glutardialdehyde led to a significant reduction or even complete inhibition of root caries [31]. A study by Walter et al. evaluated the ability of different adhesive systems to inhibit in vitro caries formation. Changes in groups where One-up Bond F (Tokuyama), iBond (Heraeus Kulzer) and Gluma Comfort Bond + Desensitizer (Heraeus Kulzer) preparations had been applied were significantly lesser than in the control group [32].

The aim of the study by Thome et al. was to analyse the inhibitory effect of restorative materials containing the antibacterial monomer 12-methacryloyloxydodecylpyridinium bromide (MDPB) in vitro on the formation of artificially induced secondary root caries in a biological model. The study showed that MDPB-containing composites inhibit caries progression regardless of the bonding system used, but MDPB-containing composites were not shown to perform better than conventional glass ionomers [33].

Yttrium is chemically a rare earth element which finds many applications in medicine. It is most commonly used in medical lasers and biomedical implants, and in dentistry as an antibacterial, anticarious, and contrast agent for X-ray imaging [34]. Yttrium is a marker for X-ray microanalysis, while as the yttrium fluoride compound it has a remineralising, cariostatic effect and protects tissues from formation of secondary caries, interferes with biofilm formation and weakens carbohydrate metabolism of plaque bacteria.

Dental restorative materials which are impermeable to X-rays enable detection of secondary caries by means of X-ray analysis, which is the cause of up to half of all dental procedures in adult patients. In addition, X-ray impermeable materials allow to assess quality of the restoration by means of X-ray imaging by revealing blisters in the material, overhangs, correct shape of the restoration, presence of restored contact points. X-ray imaging is expected to become one of primary diagnostic aids for dentists in patient examination. Poorterman et al. [10] reported that clinical examination detects <15% of leaking fillings, while the rest were found radiographically [35,36].

Moshabab et al. conducted a study to evaluate adhesion strength and antibacterial activity of a conventional orthodontic composite resin mixed with yttrium fluoride nanoparticles (YF₃). Yttrium fluoride nanoparticles were added to a conventional orthodontic composite resin (Transbond XT) at concentrations of 1%, 2%, and 3%. The results suggested that yttrium fluoride nanoparticles mixed with a conventional resin at a concentration of 1% showed significant antimicrobial activity and did not impair the adhesion strength [37].

Researchers are looking for ways to improve imaging of infiltrant penetration by enriching them with compounds that also have potential antimicrobial activity. In an ex vivo study, Kielbassa et al. investigated the effect of nanosilver particles added to a conventional infiltration resin (Icon) on external penetration into natural proximal enamel and dentin caries after internal tunnel preparation and internal infiltration, as compared to a standard infiltrant. The introduction of silver nanoparticles had no effect on external penetration of the resin. There were no significant differences between the groups in terms of internal or external infiltration and the areas of non-infected lesions were not significantly different (*p*> 0.109; *t*-test) [38]. The choice of silver as a marker for microscopic observations and as an antimicrobial agent may be puzzling due to its allergenic properties [39].

Researchers are trying to find a wider application for the method of dental hard tissue infiltration. A study by Lausch et al. evaluated three treatment methods for fissure caries occurrences without a cavity in terms of penetration ability: an infiltrant, an infiltrant with a microfiller, and an infiltrant–sealer combination—first infiltration, then fissure sealing. It was deduced from the study that infiltrant with a microfiller appears to be suitable for filling fissures and cavities as with a fissure sealer and that after an experimental etching regime-etching with 15% hydrochloric acid (HCl) mixed with abrasives and 15% HCl solution (1:1)—it penetrates carious lesions similarly to a conventional infiltrant preparation [40].

Active root caries usually occurs in the gingival region—up to 2 mm from the gingival margin. Lesions located further from the gingival margin are usually inactive, which is associated with plaque buildup along the gingival margin [41]. Initial root caries spreads along the enamel–cementum junction at the root surface. More advanced root lesions progress towards dental pulp. Exposed root cementum in the cervical region is extremely thin, the critical pH of the cementum is 6.7 (critical pH of enamel is 5.5), and hydroxyapatite crystals of dentin and cementum are smaller than those located in enamel, which results in a reactive surface six times larger. That is why root caries progresses in cementum about twice as fast as in enamel tissue. Early diagnosis, treatment and prevention of root caries are very important, as extensive cavities have a poor prognosis and are a frequent cause of tooth loss. Research in this area is particularly important given the structure of our society—the World Health Organisation (WHO) expects the population of adults aged 60 and over to more than triple from 600 million in the year 2000 to 2 billion in 2050 (WHO 2011). This demographic shift has important implications for public health [26].

The difference in development of a carious process in enamel and root cementum is due to the differences in their structures. The earliest clinically observable carious lesion on enamel is a white patch (*macula alba*) which is caused by demineralization of the subsurface layer of enamel. The outer surface of enamel remains initially intact, probably due to the remineralising effect of fluoride contained in saliva. The white spot looks like a small, opaque, cloudy field, more noticeable on dry than wet enamel [42]. In root caries, destruction of cementum begins along junctions between calcified layers of outer Sharpey’s fibres and inner collagen fibres, as well as along growth lines, where Gram-positive microorganisms invade. Dentin caries is similar to crown and root caries. It involves a decrease in the lumen of dentinal tubules followed by significant demineralisation of intercanal dentin, as well as destruction of closed tubules and peri-canalicular dentin. The possibility of remineralisation in enamel and dentin was described, as a significant demineralisation gradient was observed in enamel and dentin caries before bacterial invasion. In cementum, simultaneous destruction of mineral and organic components seemed to occur [43,44]. Cementum in the cervical area is a thin layer of tissue less mineralised than enamel, which, when exposed due to recession or periodontal disease, is quickly destroyed, making it easy for the carious process to penetrate dentin. Therefore, it is imperative to prevent root defects, disclose them early, and treat them. It is also important to look for advanced, minimally invasive methods of initial root caries treatment.

The use of 4% concentration of yttrium trifluoride did not facilitate microscopic observations by the authors of the current research due to high agglomeration of particles, which is a result of higher interaction of intermolecular forces. This was confirmed by a previous study by M. Skucha-Nowak et al. who used sonicated yttrium trifluoride as a tracer to obtain grains less than 1 μm in diameter. Microscopic observations of demineralised enamel subjected to infiltration showed that ytterbium trifluoride nanoparticles tend to aggregate (combine) and form larger particles, which is unfavourable for a dental infiltrant [20].

The null hypothesis was partially confirmed by the study. The use of yttrium trifluoride (2%) facilitates observation in a Hitachi S-3400N scanning electron microscope. However, it is not crucial, as infitrant penetration is observable in cementum and root dentin tissues, in contrast to enamel tissue [20]. The depth of tissue infiltration ranged from 80 to 120 μm, which agrees with the study of Kielbassa et al. who determined the depth of infiltration of tooth tissue to be approx. 100 μm [10].

## 5. Conclusions

On the basis of the carried-out research, the following conclusions may be drawn:

1. There were no differences between the degree of penetration of an experimental preparation with the characteristics of a dental infiltrant, as compared to a commercial preparation.
2. The use of a higher concentration of yttrium trifluoride (4%) as a tracer leads to agglomeration of its molecules which hinders penetration of the resulting agglomerates into the decalcified tooth tissue.
3. Penetration of the infiltration preparation can be observed in cementum and dentine tissue within the root, even without an indicator. The use of 2% yttrium trifluoride facilitates the observation, yet it is not absolutely necessary.

## 6. Patents

Experimental preparation patent was reported in 2021 under number P.439851.

## Data Availability

The data presented in this study are available from the corresponding authors.
